# Red Blood Cell Variables in Children and Adolescents regarding the Age and Sex

**Published:** 2019-04

**Authors:** Jasmina PLUNCEVIC GLIGOROSKA, Serjoza GONTAREV, Beti DEJANOVA, Lidija TODOROVSKA, Daniela SHUKOVA STOJMANOVA, Sanja MANCHEVSKA

**Affiliations:** 1. Institute of Physiology and Anthropology, Faculty of Medicine, University Ss Cyril and Methodius, Skopje, Republic of Macedonia; 2. Faculty of Physical Education, Health and Sport, University Ss Cyril and Methodius, Skopje, Republic of Macedonia

**Keywords:** Red blood cells, Hemoglobin, Hematological indices, Adolescent, Anemia

## Abstract

**Background:**

This study aimed to assess the basic red blood cell variables and hematological indices in children and adolescents and analyze the differences regarding age and sex.

**Methods:**

Overall, 320 young participants, age 8 to 18 yr, were enrolled at Laboratory of Sport’s Medicine, Medical Faculty, Skopje, Macedonia in 2016. Capillary blood samples were drawn and following hematologic parameters were measured: the red blood cell count (RBC), hemoglobin concentration (Hb), hematocrit level (Hct) and hematological indexes: mean corpuscular volume (MCV), mean hemoglobin concentration (MCH), mean corpuscular hemoglobin concentration (MCHC) and red cell distribution width (RDW).

**Results:**

RBC variables in male group showed high statistical level of significance between age different groups (*P*=0.001) for all studied parameters except MCHC (*P*=0.423) and RDW (*P*=0.174). ANOVA test and multivariate tests in female group showed that there was no significant difference for all hematological parameters between age different groups. Regarding the sex differences, male participants had significantly higher red blood count (*P*<0.001), hemoglobin content (*P*<0.001) and hematocrit (*P*<0.001).

**Conclusion:**

Hematological parameters in adolescent as inhomogeneous population are not quantified sufficiently, especially hematological indices. RBC variables, regardless of the age, differ very much between male and female examinees, in favor of the male examinees. Hematological indices were insignificantly higher in males. Regarding the age of examinees, RBC variables showed significant inter-groups differences only within male adolescents. While with girls, ages span 8 to 18 yr, we did not find significant differences for most of the hematological variables.

## Introduction

Having excellent knowledge of the referent values of red blood cells (RBCs) variables with children and adolescents is profoundly important for proper interpretation of the results of complete blood count. Reference values for RBCs variables are lower with children in comparison with the adults (1). Several studies which investigated hematologic parameters have been done in different populations, racial, ethnic and gender subgroups, even in different seasons (2–5). In most of these studies, age, ethnic and sex differences were significant and therefore it was stressed the need for establishing normal reference values for different populations.

RBC variables are fairly stable through adult life, but significant differences exist in the pediatric population. The newborn infant, older child, and adult show profound differences (6). Because hemoglobin level and red cell indices vary with age, it is crucial to take as reference standards that change in each period of life, from fetal life to adolescence. Adult value will be reached gradually during the second part of childhood, around 15 yr of age (7). To ensure that interpretation of hematology results in children are appropriate, the laboratory has to have established age-specific reference ranges (8).

The sex differences in hemoglobin level in adults are well documented, and the underlying mechanisms are probably a direct effect of sex hormones, both estrogen and androgens on erythropoiesis (9). “In pre-pubertal humans no major differences can be found between the sexes in red blood cell count or hemoglobin and serum ferritin concentrations” (10). “The difference in hematological variables between sexes emerges after onset of menstruations and persistent until 10 yr after the menopause” (9, 10). Menstruation and nutritional intake are principal reasons for lower values of hemoglobin and iron of women regarding men (11).

The total amount of hemoglobin increases more in boys than in the girls in the period of puberty (12). Among children 6–14 yr old the values increased from about 12 to about 14 gr per 100 ml of blood. In girls between 14 and 20 yr of age, the hemoglobin values decreased slightly, reaching 13gr/100ml. In boys of corresponding ages, there was an increase to about 15gr/100ml. In both sexes, these values were attained at about 20 yr of age and remained characteristic of the third decade of life (13).

A few comparative studies have been conducted on children in pre-adolescent and adolescent years and the lack of studies and information on hematological parameters for this population is obvious. Assessment of RBC variables in young population and determination of normal values is necessary for identification of anemia.

The aim of this paper was to determine the values of RBC variables with young population from both sexes, within age span 8 to 18 years. Possible differences in the group(s) have to be determined regarding the age difference and between the groups regarding the sex.

## Methods

### Subjects

Study participants consisted of 300 healthy young individuals (age span 8 to 18 yr) which participated continuously in different kinds of sports activities and were involved in regular medical pre-participation check-ups in 2016. A group with male subjects was composed of 240 participants and female group was composed of 80 participants. Both groups were divided into subgroups regarding the two-year interval: under 10 (U10); under 12 (U12); under 14 (U14); under 16 (U16); under 18 (U18).

### Blood collection

The hematological testing was part of complete medical checkup for sports pre-participation screening, during morning hours (from 8:00 to 12:00 am) in a controlled laboratory with constant temperature (between 20 °C and 24 °C) and humidity. To determine the blood count blood samples were collected from capillary vessel using sterile plastic containers with anticoagulant (EDTA K3) incorporated in its walls. An experienced evaluator was in charge of the collection procedures. Analysis was determined by automated hematology analyzer ABX Micros 60-OT (ABX hematology, Montpelier, France). The technical error intra rater measurement showed values lower than 1%. Reagents, calibrators, and controls were obtained from the instrument manufacturer. Analysis of samples was performed immediately after blood drawing. The testing was conducted at The Institute of Physiology, Medical Faculty Skopje, Republic of Macedonia.

### Definitions of analyzed hematological parameters (14, 15)

The erythrocyte or red blood counts also referred to as “RBCs”, involve counting the number of RBCs per unit volume of whole blood. Male 4.7 – 6.1 × 10^6^ cells/mm^3^; female 4.2–5.4 × 10^6^ cells/mm^3^.

1. Hematocrit (Hct) is the percentage of blood that is represented by the red blood cells. Normal ranges for hematocrit are strongly dependent on the age, and well described from newborns to adult age. Optimal values for adult males are between 42% and 54% and for female 38% to 46%.
2. Hemoglobin level (Hb) is expressed as the amount of hemoglobin in grams per deciliter of whole blood. Adult males should have between 14 to 18 g/dl, adult woman 12 to 16 g/dl.
3. Mean corpuscular volume (MCV) is the mean volume of all the red blood cells in the sample or the average size of the red blood cell. It can be calculated by dividing the hematocrit (volume of all RBC) by RBC number. The value is expressed in volume units, femtolitres (fL=10^−15^ L). The normal range is 80-94fL.
4. Mean corpuscular hemoglobin (MCH) represents the mean mass of hemoglobin in the one red blood cell and is expressed in the mass unit, picograms (pg= 10^−12^ gr). It is calculated by dividing the total mass of hemoglobin by the number of red blood cells. The normal range is 27–31 pg.
5. Mean corpuscular hemoglobin concentration (MCHC) is the mean concentration of hemoglobin in the red cell or average concentration of hemoglobin in one liter of red blood cells. It is calculated by dividing the hemoglobin by the hematocrit. MCHC fulfill the meaning of MCH considering the size of the cell. The normal range is 31.5–35 g/dl.
6. RDW or red cell distribution width is parameter that measures variation in red blood size or red blood cell volume. The reference ranges for RDW for adult is 11.6%–14.6%.

### Ethics

Institutional ethical approval was received from the Ethics Committee of the Medical Faculty, Ss Cyril and Methodius University, Skopje, Republic Macedonia (No=03-1197/5). Informed consents were obtained from the parents.

### Statistical Analysis

Statistical analysis as performed using the computer software SPSS for Windows version 14.0 (SPSS Inc., Chicago, USA). Analysis of variance factorial analysis and post hoc multiple comparisons were used to evaluate the significance of the differences. Differences in proportions were analyzed using the Chi-square test or Fisher’s exact test when appropriate. All data were presented as mean (±SD). Results were considered to be statistically significant when *P*-value was less than 0.05 (*P*<0.05).

## Results

### Hematologic parameters in males

The mean value and standard deviations for general features (age, height and weight) and hematologic parameters (RBCs-red blood cells; Hb-hemoglobin, Hct – hematocrit; and hematological indices: MCV, MCH, MCHC and RDW) for group of male participants (N=240) are presented in Table 1. All parameters are shown for five age different subgroups. High statistically significant difference is found for all general features of participants: age, height, and weight.

**Table 1:** General characteristics and hematologic parameters of the male participants (8–18 yr, N=240) for age different subgroups

***MALE***	***U10***	***U12***	***U14***	***U16***	***U18***	***P-value***
***N***	***32***	***67***	***71***	***42***	***28***	
Age(yr)	9.24 (0.32)	11.08 (0.58)	13.28 (0.28)	14.93 (0.54)	16.78 (0.35)	0.001
Height (cm)	131,78 (22.9)	148,69 (22.9)	168,08 (8.6)	177,06 (6.87)	182,29 (6.88)	0.001
Weight (kg)	33,00 (6.9)	41,93 (6.99)	58,65 (12.3)	65,68 (17.02)	76,13 (9.45)	0.001
RBC (10^9/^/dl)	4,79 (0.38)	4,84 (0.39)	5,08 (0.37)	5,27 (0.36)	5,22 (0.35)	0.001
Hb (gr/dl)	12,95 (0.89)	13,31 (0.9)	14,35 (1.15)	14,96 (0.9)	15,25 (0.98)	0.001
HCT (%)	40,48 (2.67)	40,87 (2.69)	44,02 (3.5)	46,25 (2.83)	46,79 (2.81)	0.001
MCV (μm^3^)	84,00 (3.2)	84,45 (3.2)	86,75 (3.4)	87,88 (3.5)	89,7 (2.37)	0.001
MCH (pg)	27,07 (1.36)	27,62 (28.3)	28,28 (1.5)	28,46 (1.89)	29,24 (1.45)	0.001
MCHC (g/dl)	32,21 (0.95)	32,65 (9.5)	32,61 (1.5)	32,39 (1.37)	32,62 (1.37)	0.423
RDW (%)	9,9 (0.56)	9.67 (0.6)	9,76 (0.48)	9,78 (0.45)	9,63 (0.39)	0.174

Values are mean (SD): RBC-red blood cell count, Hct,- packed cell volume, Hb - hemoglobin concentration, MCV- mean corpuscular volume, MCH- mean corpuscular hemoglobin, MCHC - mean corpuscular hemoglobin concentration, RDW- red cell distribution width

ANOVA test and multivariate tests (Pillal’s trace, Wilks Lambda, Hotelling’s trace, and Roy’s largest root) showed high statistical level of significance between age different groups (*P*=0.001) for all studied parameters except MCHC (*P*=0.423) and RDW (*P*=0.174). Post hoc multiple comparisons tests for hematologic parameters between age different groups showed that subjects from U10 and U12 groups have similar values between themselves and significantly lower values from all other groups for all parameters except for MCHC and RDW. The similar situation is within U16 and U18 group. They have insignificantly different results between themselves (for RBC, Hb, Hct, MCV, MCH) and significantly higher mean values for these parameters from the younger groups. The subjects from U14 group showed statistically higher means for hematological parameters than U10 and U12 group, but statistically lower means than U16 and U18 group.

Values are mean (SD): RBC- red blood cell count, Hct,- packed cell volume, Hb - hemoglobin concentration, MCV- mean corpuscular volume, MCH- mean corpuscular hemoglobin, MCHC - mean corpuscular hemoglobin concentration, RDW- red cell distribution width

### Hematologic parameters in girls

The mean values and standard deviations for general features and hematologic parameters for group of female participants are presented in Table 2. All parameters are shown for five age different subgroups. ANOVA test and multivariate tests showed that there is no significantly difference for all hematological parameters between age different groups (*P*>0.05). Multiple comparisons test for hematologic parameters between age different groups showed that only subjects from U10 and U18 groups significantly differ for only two parameters, RBC (*P*=0.05) and MCH (*P*=0.28). The youngest group show significantly higher mean RBC than the oldest group.

**Table 2:** General characteristics and hematologic parameters in female participants (8–18 yr, N=80) for age different subgroups

***Variable***	***U10***	***U12***	***U14***	***U16***	***U18***	***ANOVA, P***
***N***	***10***	***18***	***18***	***20***	***14***	
Age(yr)	9.11 (0.42)	10.98 (0.56)	13.12 (0.25)	14.73 (0.51)	16.81 (0.45)	0.001
Height (cm)	133,87 (9.5)	149,34 (10.8)	162,46 (6.3)	164,71 (4.3)	170,25 (7.1)	0.001
Weight (kg)	32,0 (8.29)	44,78 (10.3)	51,85 (7.86)	58,18 (9.3)	62,94 (13.4)	0.001
RBC (10^9/^/dl)	4,99 (0.39)	4,71 (0.22)	4,71 (0.46)	4,68 (0.55)	4,59 (0.26)	0.349
Hb (gr/dl)	12,98 (1.26)	13,33 (1.0)	13,08 (1.38)	12,92 (1.1)	13,49 (1.5)	0.800
Hct (%)	40,61 (3.53)	40,50 (3.2)	40,72 (3.75)	40,6 (3.4)	41,27 (3.33)	0.990
MCV (μm^3^)	81,75 (6.86)	86,25 (34.3)	82,43 (17.5)	87,0 (7.0)	89,75 (3.85)	0.362
MCH (pg)	26,15 (2.74)	27,98 (2.1)	28,25 (3.43)	27,62 (3.16)	29,32 (2.08)	0.253
MCHC (g/dl)	31,94 (0.97)	32,69 (1.3)	30,59 (6.2)	30,18 (6.38)	32,61 (1.12)	0.490
RDW (%)	9,94 (0.58)	9,89 (0.65)	9,82 (0.88)	10,15 (0.78)	10,16 (0.61)	0.712

### Comparison of red blood cell parameters by sex

The comparison of the hematologic parameters for total male and female group is presented in Table 3. All studied parameters, except MCV and MCH, showed sex-related differences. Analysis of variance factorial analysis applied to the whole male and female groups showed that male participants have significantly higher red blood count (*P*<0.001), Hemoglobin content (*P*<0.001) and hematocrit *(P*<0.001). No differences were found for mean corpuscular volume (MCV) and mean concentration of hemoglobin in one red blood cell (MCH), (*P*=0.292; *P*=0.563). MCHC, mean corpuscular concentration of hemoglobin in 1 L of RBC was significantly higher in boys (*P*=0.002) and RDW, the range of red blood cells width distribution were significantly wider in girls (*P*=0.004).

**Table 3:** Comparison of hematologic parameters of physically active boys (N=240) and girls (N=80)

***Variable***	***groups***	***mean***	***SD***	***SE***	***95% Confidence Interval for Mean***			
					***Lower Bound***	***Upper Bound***	***f***	***sig***
RBC (10^12^/dl)	boys	5.02	0.42	0.274	4.97	5.08	24.450	0.001
	girls	4.72	0.41	0.526	4.62	4.83		
Hb (gr/dl)	boys	14.08	1.29	0.084	13.92	14.25	25.617	0.001
	girls	13.15	1.19	0.155	12.84	13.46		
Hct (%)	boys	43.37	3.85	0.249	42.88	43.86	23.622	0.001
	girls	40.69	3.33	0.437	39.82	41.57		
MCV (μm^3^)	boys	86.27	4.03	0.261	85.75	86.78	1.115	0.292
	girls	85.40	9.87	1.274	82.85	87.95		
MCH (pg)	boys	28.07	1.83	0.118	27.84	28.31	0.335	0.563
	girls	27.90	2.84	0.374	27.15	28.65		
MCHC (g/dl)	boys	32.53	1.23	0.079	32.38	32.69	9.978	0.002
	girls	31.51	4.37	0.574	30.36	32.66		
RDW (%)	boys	9.75	0.49	0.319	9.68	9.81	8.496	0.004
	girls	9.98	0.72	0.943	9.79	10.17		

Values are mean, SD-standard deviation, SE-standard error.

The frequency of hemoglobin concentration is fewer than the lower boundary in nominal values, 12g/dl. With the boys, 4.6% showed subnormal values, i.e. the rest 95.4% used to have normal values. Frequency of suboptimal values of Hb with girls (13.3%) was significantly higher than with the boys (*P*=0.013) (Table 4).

**Table 4:** Frequency of normal and low hemoglobin concentration in boys and girls

***Variable***		***Hb lower than 12g/dl***	***Hb normal values***	***Total***	***Chi-square test (Pearson)***
Male	Count %	11	229	240	0.013
		4.6%	95.4%	100%	
Female	Count %	8	52	60	
		13.3%	86.7%	100%	
Total	Count %	19	281	300	
		6.4%	93.6%	100%	

## Discussion

In the Republic of Macedonia, there are no elaborate studies used as local reference ranges for basic RBC parameters and hematological indices for young population. The goal of this study was to help the physicians in comparing the laboratory test results with locally generated RBC variables values. The results of the present study support findings reported by number of authors that the red blood cell variables undergo age different changes in adolescents, and sex-related difference between boys and girls.

### Dependence of the hematologic parameters of age

Children’s reference ranges for routine hematological testing are usually stratified as reference values for newborn at birth, at 2 wk, 4 wk, 2–6 months, 6 months to year, 1 to 6 yr and 6–12 yr, for both sexes. Reference values for children older than 12 yr are different for male and female subjects (16). Some authors suggest different reference values for hematologic parameters for girls and boys after 13 yr of age (17). In this paper we decided to divide the examinees from 8 to 18 yr of age, into age different groups from 2-year intervals, groups.

The mean values for RBC, Hct, Hb, MCV and MCH in male group showed tendency of increasing with the growth of the age. These parameters in groups U10 and U12 show significantly lower values than other (older) groups, and U16 and U18 show higher values for these parameters from other (younger group). Therefore, the U14 group has significantly higher values for most of the hematologic indices than U10 and U12, and significantly lower values than two older groups U16 and U18. These data indicate that boys older than 12 but younger than 14 year of age, are in the intermediate period regarding the hematological parameters.

As far as the hematological indices with the male participants are concerned, the average size of erythrocyte (MCV) grows with the age. Average content, mass of hemoglobin in one erythrocyte (MCH), also grows gradually, with significantly highest values with U18, but with boys younger than 12 (U10 and U12) and boys older than 12 (U14, U16 and U18) there is a significant difference. The average concentration of hemoglobin in one erythrocyte (MCHC) does not show intergroup difference because the size of the cell is taken into consideration. The explanation is simple, with the age of the young examinees, the size of the cell grows and the average content of hemoglobin in it. But their ratio, i.e. concentration of hemoglobin in the cell remains approximately the same. Another parameter, RW, which describes the span of the size of different erythrocytes shown in percentages, does not show mutual difference which leads to equality of the size of erythrocytes in all different groups.

The analysis of the hematological parameters with the girls showed that there was not statistically significant difference among the examined hematological parameters. Even though there was a significant difference in age, weight and height, there was not any significant difference found among the hematological parameters, except for the RBC and MCH between the youngest and the oldest group. When age different groups were compared, the only significant difference was found in the number of the erythrocytes (RBC) between the youngest (U10) and the oldest (U18) groups and it was in favor of the younger examinees. The amount of hemoglobin is similar in all groups (it increased insignificantly with the age), but the number of erythrocytes decreases insignificantly with the age and it is much bigger with U10 then with U18. The lack of this examination is that a fairly small number of girls were included, because of the low presence of girls as patients in our laboratory.

A similar cross-sectional study of hematologic parameters was conducted in Spain where adolescents with age ranging from 13 to 18.5 yr old. Younger male subjects presented lower RBC, Hb, Hct and MCV mean values than their older counterparts. Same as in our study, these differences were not found in female subjects. As expected and as we found in this study, RBC, Hb, and Hct mean values were found significantly higher in males than in the females (18). Evaluation of hematologic indices in healthy Ugandan population (aged 1 to 94 yr) showed that erythrocytes, hemoglobin, hematocrit levels and mean corpuscular volume all significantly increased with age (*P*<0.001) and were independent of age until the age of 13 yr (*P*<0.001) (19). The hematologic parameters in youth national soccer teams were investigated and found no difference between U14, U15, and U16 groups, except for the RBC variable (20). Hemoglobin contents and the RBCs gradually rise to adult levels by the age of puberty (21). Investigation of hematological parameters in population 1 to 14 yr of age in Bangladesh showed difference between age groups and no difference was found between two sex groups (22).

### Dependence of hematologic parameters of sex

Men and women have different mean hemoglobin levels in health in venous blood — women have mean levels approximately 12% lower than men. Since no difference is noticed in the level of erythropoietin with different sexes, the difference in the intensity of erythropoiesis comes from the physiological changes in the kidneys not in bone marrow (9). There is no evidence showing reduced cellular mechanisms for hem synthesis in women, and there is no difference in the iron absorption between women and men (23). The established reference ranges for woman are under the influence of large proportion of those with iron deficiency. (11). The difference in hemoglobin concentration regarding sex has not been found in infants and preschool children (24), but it has been shown in teenagers and adolescents (25). In our research, we compare the hematological parameters with male and female examinees, as well as whole groups regardless of their age. The male examinees showed significantly higher values of RBC (5.02 *10^12^/l vs 4.72 *10^12^/l; *P*<0.001); higher values of Hb (14.08 g/dl vs 13.15 g/dl; *P*<0.001); Hematocrit (43.37% vs 40.69%; *P*<0.001). The average size of red blood cells (MCV) and medium content of Hb in them (MCH) insignificantly higher with the boys. Due to the similar size of the cells and higher total amount of hemoglobin with the males, MCHC, concentration of Hb in erythrocyte, is higher with the boys (*P*=0.002). The size span of erythrocytes is wider with the girls which lead to bigger variability of the erythrocytes size.

For the clinical reference values of RBC, Hb, and Hct no sex differences were observed bellow the age of 12. The values for males were significantly higher than in females in the age range 13–79 (26). Unusual results are reported regarding the hematologic indices in male and female children younger than 12 yr. Mean Hb, Hct, MCV, and MCH of school-aged boys were significantly lower than girls (27). In the survey on haemoglobin level in the different age groups in man and woman in Indian population in the group aged 12 to 19 yr, males showed Hb mean concentration of 11.76 g/dl and female showed higher mean value, 12.31 g/dl (28). The research on hematological indices in in Kuwaiti children aged 7–12 yr, were RBC=4.78±0.42; Hb=127.3±9.4 /dl for boys and RBC=4.7±0.4; Hb=126.9±9.8 /dl for girls. Same parameters for older children, 13–17 yr, were RBC=5.18±0.48; Hb=145±14.4 /dl for boys and RBC=4.68±0.43; Hb=129.6±9.8 /dl for girls (29). As we can see these results are concordant with ours, regarding the sex differences (in favor of boys), and regarding the existing substantial age difference in male group and no age difference in female group.

Normal hemoglobin levels according to WHO for children aged 5–12 yr above 11.5 gr/dl and teenagers aged 12–15 yr equal or above 12g/dl. Above 15 yr the adolescent are referred to as adults, and normal Hb level for adult male is 13.8–17.2 g/dl, and for adult female 12.1–15.1 g/dl (30).

The primary aim of analyzing red blood cells variables is to discover and diagnose type of anemia in case it is present. Anemia was defined as hemoglobin concentration <11g/dL for children aged between 6 and 59 months, while 11.5 g/dl for children aged 5 and 11 yr and < 12 g/dl for children older than 12 yr according to WHO (30). As a lower boundary of nominal values for hemoglobin concentration in our laboratory is considered the value of 12 g/dl. In our laboratory that value is the same for both children and adults. Only 4.6% of our male examiners had low values of hemoglobin, and much more in the female group 13.3%.

## Conclusion

The young male examinees there exists a significant difference among different age groups, with special emphasis that hematological variables in boys aged 12 to 14 yr have the intermediate values between those with pre-puberty (<12 yr) towards those adolescent boys (>14 yr). RBC variables with girls from different age subgroups did not show significant differences. Significant difference is found only in red blood cell counts between the oldest (U18 group) and youngest (U12 group) in favor of the younger girls. RBC variables, regardless of the age, differ very much between male and female examiners, in favor of the male examinees. Hematological indices were insignificantly higher in males.

## Ethical considerations

Ethical issues (Including plagiarism, informed consent, misconduct, data fabrication and/or falsification, double publication and/or submission, redundancy, etc.) have been completely observed by the authors.
